# Using the Italian Surveillance System (PASSI) as a Model to Track Health Conditions and Risk Behaviors in Corsica

**Published:** 2008-06-15

**Authors:** Pascal Vignally, Sandro Baldissera, Nancy Binkin, Jean Arrighi, Jean Pierre Amoros

**Affiliations:** Italian National Institute of Health, National Center for Epidemiology, Surveillance and Health Promotion, Rome, Italy; Italian National Institute of Health, National Center for Epidemiology, Surveillance and Health Promotion, Rome, Italy; Italian National Institute of Health, National Center for Epidemiology, Surveillance and Health Promotion, Rome, Italy; Corsican Observatory of Health, Ajaccio, France; University of Corsica, Unité INSERM U707, Corte, France

## To the Editor:

In their study, Zindah et al (1) show that common risk factors (e.g., obesity, poor diet, inactivity) create a major chronic disease burden in Jordan. These findings illustrate the importance of a surveillance system for behavioral risk factors and chronic diseases.

In Europe, chronic diseases are also responsible for the majority of deaths, and the most important risk factors for chronic diseases (obesity, physical inactivity, diabetes, hypertension, tobacco use, alcohol use) are few and largely preventable (2). To assess the impact of health promotion and disease prevention programs and to identify subgroups at greatest risk, the World Health Organization's 2002 report (3) recommended implementing risk factor surveillance. In the United States, the Behavioral Risk Factor Surveillance System (BRFSS) was established in the early 1980s to monitor the prevalence of health risk behaviors. In the last 20 years, several other countries have established similar surveillance systems. According to Bauman et al (4), key components influencing the successful establishment of such systems are the contributions of regional and local health staff in highlighting the value of locally relevant health risk behavior data, in garnering local resources and support, and in adapting data collection to local conditions. Other factors that characterize successful systems include flexibility and potential for adaptation, thereby balancing the need to collect comparable data across geographic areas against the unique needs of local areas.

In France, the National Institute for Economic Statistics has conducted surveys to obtain national estimates of health risk behaviors among adults. These data, however, are collected only at 5-year intervals, are not available regionally, and may not be appropriate for some regions. As a result, the system may not be particularly useful for regional health agencies that target resources to reduce behavioral risks and consequent illnesses (5).

The French island of Corsica shares common cultural and behavioral characteristics with the neighboring Italian regions of Liguria, Sardinia, and Tuscany. The European Union, through the Community Initiative for the European Regional Development Fund, promotes transnational and interregional cooperation. Specific projects are dedicated to cooperation between French and Italian border regions (6).

In Italy, a new system, PASSI (Progressi delle Aziende Sanitarie per la Salute in Italia), which adapts the BRFSS model to the local situation, is being implemented to monitor behavioral risk factors (7). We have developed a proposal for a pilot study using PASSI to track health conditions and risk behaviors in the Corsican population. Because similar data will be available for neighboring Italian regions, we will be able to compare the situation across regions and states.

The main objectives of the project are to measure health risk behaviors and preventive interventions in Corsica and to make available appropriate health indicators to regional and local policy makers. A series of activities is planned, including conducting the pilot study in Corsica; communicating results to all relevant stakeholders and promoting use of these findings for public health purposes; comparing results with those obtained from the PASSI surveillance system in Liguria, Tuscany, and Sardinia; and evaluating the feasibility of implementing an ongoing surveillance system in Corsica.
